# Endometrial pathogenic *Escherichia coli* in canine pyometra: severity of tissue damage, host defense evasion, and antimicrobial resistance profile

**DOI:** 10.3389/fvets.2026.1676990

**Published:** 2026-02-12

**Authors:** Ada Rota, Michela Corrò, Lorella Maniscalco, Elena Spagnolo, Elena Tonon, Serena Genovese, Alessia Bertero

**Affiliations:** 1 Department of Veterinary Sciences, University of Turin, Grugliasco, Italy; 2 Istituto Zooprofilattico Sperimentale delle Venezie, Legnaro, Italy

**Keywords:** antimicrobial resistance, bitch, *Escherichia coli*, phosphoinositide 3-kinase expression, pyometra, virulence factors

## Abstract

*Escherichia coli* (*E. coli*) is the bacterium most frequently isolated from canine pyometra. The Endometrial Pathogenic *E. coli* (EnPEC) show virulence traits that cause tissue damage through adhesion/invasion of cells, and toxin production. Phosphoinositide 3-kinases (PI3K) are intracellular signal transducers with a fundamental role in the innate immune defense. Bacteria that inhibit the PI3K pathway can escape the host defenses. The aim of this study was (a) to investigate the association among the characteristics of *E. coli* isolated from pyometra (phylogroup, hemolysis, CNF—cytotoxic necrotizing factor, CDT—cytolethal distending toxin), the severity of tissue lesions and the expression of PI3K; (b) to assess the resistance profile. *E. coli* was isolated from 17/21 uteri, always in pure culture. All *E. coli* belonged to the phylogroup B2 and 12/17 uteri contained hemolytic strains. CNF was evidenced only in the hemolytic strains. CDT was never detected. Four uteri showed perforation: all contained CNF-positive hemolytic *E. coli* and PI3K expression was negative. No significant association was found between the characteristics of *E. coli* and the degree of tissue damage or PI3K expression. The non-hemolytic *E. coli* tended to show higher resistance toward some antimicrobials. Our results confirm that the highly virulent phylogenetic group B2 is often involved in canine pyometra. PI3K was not expressed in the perforated uteri, suggesting a potential interference of the pathogen with the host cells defenses. Future research should be focused on the mechanism of host defense evasion through the inactivation of the PI3K signaling pathway.

## 1 Introduction

Pyometra is an ascending bacterial infection that affects more than 20% middle-aged to elderly intact bitches (1, 2). The pus-filled uteri show macroscopical and histological alterations that can have different degrees of severity (3) and uterine wall rupture can occur. The microorganisms that are involved in the pathology belong to the host’s vaginal or intestinal microbiota (4), with the prevalence of *Escherichia coli* (*E. coli*) which is the bacterium that more frequently grows in pure culture from samples of the uterine content (5, 6). The *E. coli* strains that are isolated from uterine exudates are epidemiologically and phylogenetically distinct from those living as commensal in the intestinal tract of dogs or those that cause diarrhea and other gastrointestinal disorders (6). They are characterized by the presence of several virulence factors that promote colonization of extraintestinal sites, enhancing their ability to adhere and invade host cells and tissues, and secrete toxins that directly affect host cellular functions, increasing tissue damage (7). A toxin that can be produced by *E. coli* is the cytotoxic necrotizing factor (CNF): it can be encoded by a pathogenicity-associated island (PAI) which also comprises the genes encoding for alpha-hemolysin and P-fimbriae, or by plasmids, which can also encode the cytolethal distending toxin (CDT) (8). Virulence factors genes have been spread among bacteria by horizontal transfer and represent an adaptive advantage that allows the colonization of niches that cannot be colonized by commensal *E. coli* strains (9). *E. coli* is primarily a commensal or pathogen of the intestinal tract but a subset of strains has the ability to cause extraintestinal infections. Extraintestinal pathogenic *E. coli* (ExPEC) strains are a major cause of urinary tract infections in dogs (7, 10).

Being both a widespread gut commensal of vertebrates and a versatile pathogen, *E. coli* is recognized as a multi-sectoral indicator of antimicrobial resistance (AMR) (11). The virulence and antibiotic resistance of pathogenic strains can be promoted by the selective pressures in the habitats of commensal strains, rendering commensal *E. coli* strains reservoirs of virulent and resistant ones (9). Recent studies have highlighted the pivotal role of host-directed therapies in improving the efficacy of antimicrobial treatments, especially in the context of intracellular pathogens that evade immune clearance and antibiotic action (12). Phosphoinositide 3-kinases (PI3Ks) are a family of intracellular signal transducer enzymes that have emerged as promising therapeutic targets due to their central involvement in immune cell functions, including neutrophil chemotaxis, macrophage phagocytosis, and regulation of inflammatory responses (13, 14). Increasing evidence suggests that certain bacterial pathogens exploit PI3K signaling to interfere with phagosome maturation and prolong intracellular persistence: for instance, enteropathogenic *E. coli* (EPEC) inhibits host PI3K/Akt-dependent pathways to prevent phagocytic uptake (15). In addition to its ability to colonize and persist in the uterine environment, Endometrial Pathogenic *E. coli* (EnPEC) might then manipulate host immune responses and intracellular signaling pathways to promote survival and evade clearance.

The aim of this study was to investigate the characteristics of the EnPEC isolated from canine pyometra (hemolysis, phylogenetic groups and the virulence factors CNF and CDT) when uterine lesions are severe and, in particular, when uterine wall rupture occurs and to contribute to the understanding of host-pathogen interactions involving PI3K signaling pathway in the uterine tissues. A second purpose of the investigation was to discuss the resistance profile of the isolated EnPEC strains.

## 2 Materials and methods

### 2.1 Animals

Twenty-one female dogs that had undergone ovariohysterectomy for pyometra treatment at the Veterinary Teaching Hospital of the University of Turin during the period September 2023–December 2024 were included in this study. Inclusion criteria consisted in having carried out a bacterial culture exam of the uterine content and having collected the uterus for histological analysis.

The study obtained approval from the Ethical Committee of the Department of Veterinary Sciences of the University of Turin (Italy) (n. 0126683, 12/02/25) and written informed consent was obtained from the owners.

### 2.2 Clinical data

The following data were drawn from the clinical records: breed, age, weight, history of urinary tract infection, type of pyometra (open/closed cervix), leukogram, presence of peritonitis at the time of surgery, days of hospitalization and clinical outcome.

The phase of the oestrus cycle was deducted from the reproductive history provided by the owner and confirmed by Progesterone concentration (mini VIDAS®, bioMérieux, Marcy l’Etoile, France). Diestrus was categorized on the basis of the referred onset of the last heat and by direct ovarian and corpora lutea observation at surgery: early (until 40 days after pro-oestrus onset, prominent corpora lutea); middle/late (more than 40 till 70 days after pro-oestrus onset, smaller corpora lutea or yellow-whitish small corpora lutea) (16, 17).

### 2.3 Microbiological and molecular investigations

Immediately after removal of the uterus, a bacteriological swab (ESwab 480 CE, Copan Italia Spa, Brescia, Italy) was plunged into the uterine lumen through an incision done with a sterile surgical blade. Bacterial culture (aerobic and anaerobic) and broth microdilution test were performed at the Istituto Zooprofilattico Sperimentale delle Venezie (PD, Italy), according to standard laboratory techniques (18), within 48 h of collection (19). Bacterial species identification was performed by the Bruker Microflex LT MALDI-TOF mass spectrometer (Bruker Daltonics, Milano, Italy) equipped with FlexControl software (version 3.3, Bruker Daltonics, Milano, Italy). The score for identification was ≥ 2.3. The hemolytic activity of *E. coli*, associated with the expression of *E. coli* alphahemolysin, was evaluated phenotypically on nutrient medium plates added with 5% sheep blood (Blood Agar, Biolife, Milan, Italy).

Phylogenetic group was determined by means of a Multiplex End-Point PCR and of two End Point PCR specific for phylo-group C and phylo-group E (20) performed with Faststart Taq DNA Polymerase (Merck, Darmstadt, Germany) and with primers synthetized by Metabion International (Planegg, Germany) (Supplementary material 1). DNA amplification followed this procedure: initial denaturation at 94 °C for 5 min, 30 cycles of denaturation at 94 °C for 30 s, annealing at 59 °C (57 °C for group C and 55 °C for group E) for 30 s, extension at 72 °C for 30 s and final extension at 72 °C for 5 min (Supplementary material 1).

A Multiplex PCR was used to detect the presence of virulence genes codifying Cytotoxic Necrotizing Factor (CNF) and Cytolethal Distending Toxin (CDT) (21). In detail, a Multiplex PCR Kit (QIAGEN, Hilden, Germany) and primers synthetized by Eurofins Genomics (Ebersberg, Germany) were used (Supplementary material 2). The DNA amplification was performed using the following program: initial denaturation at 95 °C for 15 min, 35 cycles of denaturation at 94 °C for 30 s, annealing at 57 °C for 90 s, elongation at 72 °C for 90 s, final elongation al 72 °C for 10 min and final hold 12 °C (Supplementary material 2). Bacterial DNA extraction for phylo-group analyses was carried out using the simple workflow protocol of MagMAX™ CORE Nucleic Acid Purification Kit (Applied Biosystem—Thermo Fisher Scientific) combined with the KingFisher Apex Robot (Thermo Fisher Scientific); while to detect the virulence genes, mechanical extraction was performed by incubating the sample at 99 °C for 10 min and centrifuging at 16,000 g for 2 min.

Antimicrobial Susceptibility Testing was performed using commercial plates (Thermo Scientific™ Sensititre™ ITISVE7, Thermo Fisher Diagnostics, Segrate (MI), Italy) specifically manufactured by TREK Diagnostic Systems Ltd. (West Sussex, RH19 1XZ, UK) for the Istituto Zooprofilattico Sperimentale delle Venezie. The quality control strain was *E. coli* ATCC 25922. Dilutions for the MICs were obtained with BBL™ Mueller Hinton II Broth- Cation Adjusted (Becton Dickinson and Company BD Diagnostic Systems, PO Box 999, Sparks Md 21152–0999 USA). The plates were read with Thermo Scientific™ Sensititre™ Vizion 436-A, Sensititre™ SWIN software 3.4 (Thermo Fisher Diagnostics, Segrate (MI), Italy). Minimum Inhibitory Concentration (MIC) was assessed for the following panel of antimicrobials belonging to different classes: Aminoglycosides (amikacin, kanamycin, gentamicin), Fluoroquinolones (enrofloxacin, pradofloxacin), Tetracyclines (tetracycline, doxycycline), Folate Pathway Inhibitors (trimethoprim sulfamethoxazole), Beta lactam/Beta lactamase inhibitor combinations (ampicillin, amoxicillin-clavulanic acid), Cephems (first generation cephalosporin: cephalexin, cephazolin; third generation cephalosporin: cefpodoxime, cefovecin). Dilution ranges are reported in Supplementary material 3. Antimicrobial breakpoints were those set by the Clinical and Laboratory Standards Institute for skin/soft tissue/urinary trac Enterobacterales/*E.coli* infections in dogs (22).

Multidrug-resistant isolates were defined as those exhibiting acquired resistance to at least one agent in three or more antimicrobial categories (23).

### 2.4 Histological analyses

Tissue samples were collected from the middle portion of a uterine horn or, when present, from the area including the perforated wall, fixed in 10% buffered formalin, embedded in paraffin, cut at 4 μm and stained with hematoxylin and eosin. Stained sections were viewed using a Nikon light microscope (Eclipse E200) and the images were captured with a digital camera (QIcam FAST 1394). The Image-Pro Premier 9.1 software was used to perform morphometric analysis.

The thickness of the endometrium and myometrium was measured at 4 randomly selected places and was averaged: the endometrium/myometrium ratio was calculated. Histologically, pyometra was categorized as hyperplastic or atrophic according to the relative thickness of endometrium and myometrium: hyperplastic when endo/myo ratio > 0.78, atrophic when much decreased (3). The percentage of endometrium occupied by endometrial gland lumina was calculated in 4 randomly selected areas of 0.5 mm^2^ to assess the cystic changes of the endometrial glands. When higher than 25%, the endometrium was defined as cystic (3). The endometrial stroma was evaluated for amount of inflammatory infiltrate (mild, moderate, severe), oedema (present or absent), hemorrhages (absent, mild multifocal, multifocal), fibroblasts proliferation (few, moderate, severe), vascular congestion (diffuse or multifocal), presence of squamous metaplasia (absent, present, severe) and necrosis (present or absent).

Immunohistochemistry (IHC) was conducted to assess the expression of PI3K (1:400 dilution; Rabbit monoclonal antibody, code 19591, Cell Signalling Technology, Danvers, MA, USA). IHC was performed on tissue sections with a thickness of 4 μm as follows. Endogenous peroxidase activity was blocked with 0.3% H_2_O_2_ for 30 min. Then, heat-induced antigen retrieval was carried out with citrate buffer at 98 °C with a pH of 6 for 30 min. The sections were then incubated with primary antibodies for 2 h at 4 °C. Detection was carried out using the Vectastain Elite ABC Kit (Vector Laboratories Inc., Newark, CA, USA) with diaminobenzidine (DAB; ImmPACT DAB from Vector Laboratories Inc., Newark, CA, USA) as chromogen. Hematoxylin was used as a nuclear counterstain. Immunopositivity was evaluated using a semiquantitative scoring system assessing distribution (0–4) and intensity (0–4) (range of the sum: 0–8; positivity >3) (24).

### 2.5 Analysis of data

Prevalence was calculated for clinical data and descriptive statistics.

The differences between the “*E. coli* pyometra” and the “pyometra with other bacteria” in relation to the age of the bitches and the number of leucocytes/neutrophils were evaluated using the t-test for independent samples. Differences in the clinical presentation of pyometra (open/closed) in the same two groups were assessed using Fisher’s exact test.

A score was assigned to each of the following histological parameters: inflammatory reaction (mild, moderate, severe: 1, 2, 3), presence of hemorrhages (absent, multifocal mild, multifocal: 0, 1, 2), fibroblast proliferation (few, moderate, severe: 1, 2, 3), PI3K expression (present/absent: 0/1). The sum was used to categorize the severity of tissues lesions from a minimum value of 3 to a maximum of 9.

The data were analysed using GraphPad Prism for macOS version 10.5.0. (GraphPad Software, LLC). The association between the characteristics of *E. coli* (hemolysis, CNF virulence factor) and the presence of uterine wall ulceration, the histological type of pyometra (hyperplastic or atrophic), the severity of tissue lesions and the expression of PI3K was assessed by means of Fisher’s exact test.

The same analysis was used to compare the percentage of resistance toward the tested antimicrobials of different *E. coli* isolates (hemolytic vs. non-hemolytic). Results were considered significant if *p* < 0.05.

## 3 Results

*Escherichia coli* was isolated from 17 out of 21 uteri (80.9%), always in pure culture. Of the other four pyometra cases in which *E. coli* was not isolated, two resulted in single culture of *Haemophilus haemoglobinophilus* and *Klebsiella oxytoca* respectively; the other two, in mixed culture, of *Streptococcus canis* and *Burkholderia cepacia* and of *Staphylococcus pseudintermedius* and *Enterococcus canintestini, respectively.*

The mean age (± standard deviation) of the bitches with *E. coli* pyometra was 9.8 ± 2.7 years, ranging from 5.0 to 14.3 years and the mean age of the other bitches was 12.0 ± 5.3 years (range 6.2–16.7 years). No significant differences were found between the two groups in terms of age. Fourteen bitches were different breeds while seven were mixed breed. History never reported that the dog had suffered from urinary tract infection.

Closed cervix pyometra accounted for 10/18 of the cases (55.6%) of cases (8/15, 53.3% *E. coli* pyometra and 2/3, 66.7% other pyometra, no significant differences between the two groups). The surgical record reported the presence of abdominal fluid and peritonitis in four bitches with *E. coli* pyometra, and of hemorrhagic-content in another bitch. The uterus showed a wall discontinuity, with tentative omental reparation, in four bitches from which hemolytic *E. coli* was isolated.

Nineteen animals were discharged from the hospital the day after surgery; one bitch that developed abdominal fluid was discharged after 7 days and a bitch that was septic at presentation required 17 days of hospitalization. A hemolytic *E. coli* was isolated in both animals.

In most bitches, the pathology developed during diestrus, more commonly in early diestrus (10/16, 62.5%) than in late diestrus (5/16, 31.2%). One bitch had ovarian cysts while the cycle stage was not reported in 5 cases.

Most bitches showed leucocytosis (26.546 ± 14.564 K/μL, mean ± SD) with neutrophilia (18.935 ± 12.447 K/μL), both when *E. coli* was involved and when other bacteria were isolated; severe neutropenia (0.940 K/μL) was reported in the single case of septic shock.

### 3.1 *Escherichia coli* virulence characteristics

In six cases, more than one *E. coli* strain was cultured from a single uterus and tested, for a total of 24 isolates: when originating from the same uterus, the isolates always showed analogous characteristics and are then grouped in Table 1.

**Table 1:** *Escherichia coli* virulence characteristics (hemolysis and CNF detection) of the strains isolated in the uteri.

N	Hemolysis	CNF
1	+	+
2	+	+
3	−	NP
4	−	NP
5	−	−
6	−	−
7	+	+
8	+	+
9	+	+
10	+	+
11	+	+
12	+	+
13	+	+
14	+	NP
15	+	+
16	+	−
17	−	−

Positive: +, negative: −, not performed: NP.

All the tested *E. coli* strains resulted to belong to the phylogenetic group B2. Twelve out of 17 uteri contained hemolytic *E. coli* strains.

CNF was evidenced exclusively in the hemolytic strains, in all isolates except one. All the tested isolates resulted negative for CDT (Table 1).

The four cases showing perforated uterine wall contained CNF-positive hemolytic *E. coli*.

### 3.2 Histological analyses and PI3K expression

In most cases (13/15, 86.7%), pyometra was categorized as hyperplastic and the endometrium was cystic in 53.3% (8/15) of uteri (Table 2).

**Table 2:** Histological characteristics of the organs: perforation of the uterine wall (perforated/intact: ±), endometrium/myometrium ratio (hyperplastic if > 0.78), % endometrial gland (percentage of endometriun occupied by endometrial gland lumina: cystic if > 25%), inflammatory reaction (mild, moderate, severe), edema (present/absent: +/-, not ±), presence of hemorrhages (absent, multifocal mild, multifocal), fibroblast proliferation (few, moderate, severe), vascular congestion (diffuse/multifocal), squamous metaplasia (absent, present, severe), necrosis (absent, focal, present), PI3K expression (distribution + intensity: 0–8).

N	Uterinewall perforation	Endometrium/myometrium ratio	% endometrial gland	Inflammatory reaction	Edema	Hemorrhages	Fibroblast proliferation	Vascular congestion	Squamous metaplasia	Necrosis	PI3K expression
1	+	0.34 (atrophic)	5.74	Moderate	−	Multifocal mild	Moderate	Diffuse	Absent	Absent	0
2	−	0.73 (atrophic)	41.42 (cystic)	Severe	−	Multifocal	Moderate	Multifocal	Severe	Absent	3 + 3
3	−	1.07 (hyperplastic)	9.78	Severe	−	Multifocal	Moderate	Diffuse	Absent	Present	3 + 2
4	−	1.33 (hyperplastic)	12.69	Severe	−	Absent	Moderate	Diffuse	Absent	Absent	3 + 2
5	−	3.21 (hyperplastic)	41.7 (cystic)	Severe	−	Absent	Few	Diffuse	Present	Absent	2 + 1
6	−	2.99 (hyperplastic)	15.24	Severe	−	Absent	Severe	Diffuse	Absent	Absent	2 + 1
7	+	1.82 (hyperplastic)	6.33	Severe	−	Absent	Moderate	Diffuse	Present	Absent	0
8	−	0.94 (hyperplastic)	26.51 (cystic)	Severe	−	Absent	Few	Diffuse	Present	Absent	2 + 3
9	+	1 (hyperplastic)	4.62	Mild	+	Multifocal	Few	Diffuse severe	Absent	Absent	2 + 1
10	−	2.35 (hyperplastic)	48.58 (cystic)	Moderate	−	Absent	Few	Multifocal	Present	Focal	2 + 3
11	−	1.49 (hyperplastic)	27.19 (cystic)	Severe	−	Absent	Few	Multifocal	Present	Absemt	2 + 2
12	−	1.16 (hyperplastic)	9.53	Moderate	−	Multifocal	Moderate	Diffuse	Absent	Absent	3 + 2
13	−	2.61 (hyperplastic)	28.77 (cystic)	Severe	−	Multifocal	Few	Diffuse	Present	Present	2 + 2
14	−	2.65 (hyperplastic)	162.78 (cystic)	Moderate	−	Multifocal	Moderate	Diffuse	Present	Absent	4 + 3
15	−	2.73 (hyperplastic)	40.57 (cystic)	Severe	−	Multifocal	Few	Diffuse	Present	Present	2 + 3

The endometrial stroma showed severe inflammation in 66.7% (10/15) of cases, multifocal hemorrhages (8/15, 53.3%), diffuse or multifocal vascular congestion (15/15, 100%), squamous metaplasia (9/15, 60%), necrosis (4/15, 26.7%). Table 2 shows the characteristics of the tissue damage observed in the individual organ, while Table 3 illustrates the categorization of the different parameters investigated: inflammatory reaction, hemorrhages, PI3K expression, and the overall assessment of the severity of tissue lesions.

**Table 3:** Categorization of the different parameters investigated: inflammatory reaction (mild, moderate, severe: 1, 2, 3), presence of hemorrhages (absent, multifocal mild, multifocal: 0, 1, 2), fibroblast proliferation (few, moderate, severe: 1, 2, 3), PI3K expression (positive/negative: 0/1) and overall assessment of the severity of tissue lesions (sum of scores assigned to each histological characteristic for each organ). The score (number in brackets) attributed to the different histological features of each sample is proportional to the severity of the lesions.

N	Inflammatory reaction	Hemorrhages	Fibroblast proliferation	PI3K expression	Overall assessment of the severity of tissue lesions
1	Moderate (2)	Multifocal mild (1)	Moderate (2)	− (1)	6
2	Severe (3)	Multifocal (2)	Moderate (2)	+ (0)	7
3	Severe (3)	Multifocal (2)	Moderate (2)	+ (0)	7
4	Severe (3)	Absent (0)	Moderate (2)	+ (0)	5
5	Severe (3)	Absent (0)	Few (1)	− (1)	5
6	Severe (3)	Absent (0)	Severe (3)	− (1)	7
7	Severe (3)	Absent (0)	Moderate (2)	− (1)	6
8	Severe (3)	Absent (0)	Few (1)	+ (0)	4
9	Mild (1)	Multifocal (2)	Few (1)	− (1)	5
10	Moderate (2)	Absent (0)	Few (1)	+ (0)	3
11	Severe (3)	Absent (0)	Few (1)	+ (0)	4
12	Moderate (2)	Multifocal (2)	Moderate (2)	+ (0)	6
13	Severe (3)	Multifocal (2)	Few (1)	+ (0)	6
14	Moderate (2)	Multifocal (2)	Moderate (2)	+ (0)	6
15	Severe (3)	Multifocal (2)	Few (1)	+ (0)	6

**Figure 1:**
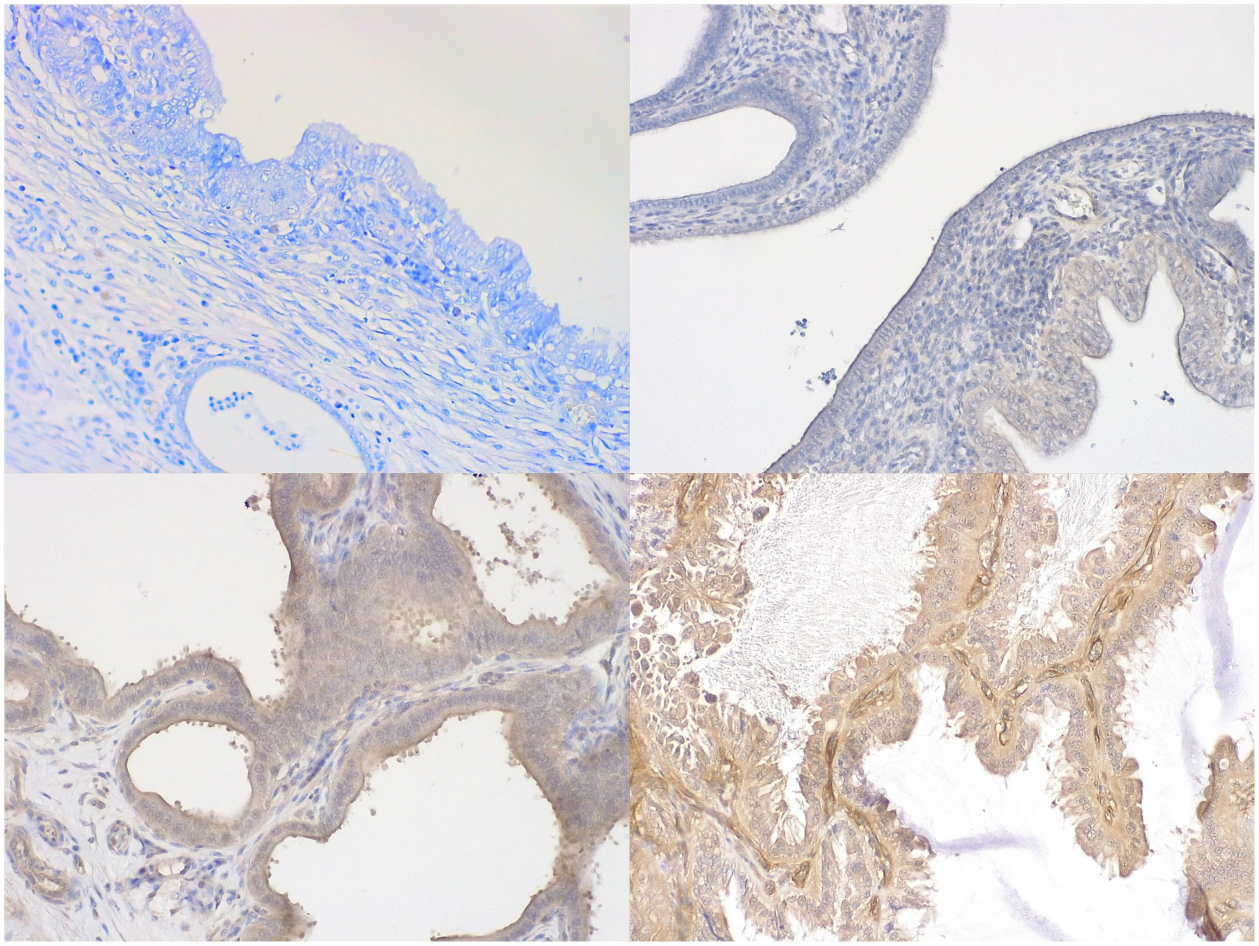
Immunohistochemical expression of PI3K in canine uteri, hematoxylin counterstaining. Top left: complete absence of immunolabeling (negative); top right: multifocal weak immunolabeling in endometrial cells (score 2 + 1); bottom left: moderate immunolabeling in endometrial cells (score 4 + 2); bottom right: diffuse and intense immunolabeling in endometrial cells (score 5 + 3). Objective 4 × .

PI3K expression ranged from 0 to 7, resulting zero in two uteri, one atrophic and one hyperplastic pyometra case, both perforated. Low intensity and scattered distribution of positivity was found in a third perforated uterus (Figure 1, top right) with hyperplastic wall, and in other two hyperplastic uteri (Table 2). Unfortunately, the fourth perforated uterus could not be analysed. Figure 1 shows different degrees of PI3K expression in the uterine wall.

No significant association was found between the characteristics of *E. coli* (hemolysis and CNF detection) and the clinical or histological characteristics of the pyometra cases.

### 3.3 *Escherichia coli* resistance profile

The percentage of resistance of the *E. coli* isolates toward the 14 tested antimicrobials, based on the dog skin/soft tissue breakpoints for Enterobacterales when available or on dog urinary tract breakpoints for *E. coli* (22), is shown in Table 4, together with the MICs. All the isolates are resistant to ampicillin, amoxicillin-clavulanic acid and cephalexin, Fluoroquinolones resistance is relatively low with 4/24 (16.7%) *E. coli* isolates showing resistance to enrofloxacin and 2/24 (8.3%) to pradofloxacin.

**Table 4:** MIC values (mg/L) of the tested antimicrobials for *E. coli* isolates grouped according to hemolytic phenotype. Data are presented as MIC range (min–max), median, and as resistant isolates [*n* (%)]. Interpretation of MICs (S/I/R) was based on the dog skin/soft tissue breakpoints for Enterobacterales when available or on dog urinary tract breakpoints for *E. coli* (22).

Antibiotic	Hemolytic *E. coli* (*N* = 16)			Non-hemolytic *E. coli* (*N* = 8)		
	MIC range	Median	*n* (%) *R*	MIC range	Median	*n* (%) *R*
Amikacin	<1–8	2	1 (6.25)	2–8	3	1 (12.5)
Kanamycin	–	<8	0	–	<8	0
Gentamicin	<0.5 – >16	<0.5	1 (6.25)	<0.5–2	1	0
Enrofloxacin	<0.06–0.5	<0.06	1 (6.25)	<0.06 – >4	<0.06	3 (37.5)
Pradofloxacin	–	<0.12	0	<0.12 – >2	<0.12	2 (25)
Doxycycline	0.5–8	2	16 (100)	0.5–4	1.5	8 (100)
Tetracycline	<2–4	<2	0	<2–4	<2	0
Trimethoprim sulfamethoxazole	<0.5/9.5–1/19	<0.5/9.5	0	–	<0.5/9.5	0
Amoxicillin-clavulanic acid (SST)	2/1–8/4	4/2	16 (100)	2/1 – >32/16	4/2	8 (100)
Ampicillin (SST)	2 – >32	4	16 (100)	1 – >32	6	8 (100)
Cephazolin	1–8	2	5 (31.25)	2 – >32	4	5 (62.5)
Cephalexin (SST)	4–16	8	16 (100)	8 – >32	16	8 (100)
Cefpodoxime	<0.5–1	<0.5	0	<0.5 – >8	1	2 (25)
Cefovecin	0.25–4	1	1 (6.25)	0.12 – >8	1	2 (25)

No multidrug-resistant strains were detected.

The comparison between hemolytic and non-hemolytic strains showed that the percentage of resistance toward enrofloxacin tended to be higher in the latter (1/16, 6.25% *vs* 3/8, 37.5%, *p* < 0.09). Non-hemolytic isolates may have a more resistant profile, including partial resistance to fluoroquinolones and third generation cephalosporins, although no statistically significant differences were found in terms of resistance to the antibiotic panel tested, probably due to the small number of samples.

## 4 Discussion

The link between canine pyometra and *E. coli* is well known since it is the bacterium most frequently isolated from uterine exudates (1, 5) and in our work it was isolated in pure culture in over 80% of uteri. *E. coli* is a widespread gut commensal of vertebrates but also a versatile pathogen that causes both intraintestinal and extraintestinal diseases (25). Extraintestinal pathogenic *E. coli* (ExPEC) typically occupy a niche in the intestinal microbiota and emerge from this reservoir to cause extraintestinal infections (26). ExPEC show virulence traits that result in tissue damage through adhesion and invasion of cells, and direct secretion of toxins that affect cellular functions (7). EnPEC are a pathotype that belongs to the ExPEC group, and they are isolated from animal and human endometrial infections (27).

Although *E. coli* is the most frequent bacterium isolated from canine pyometra (1), genetic studies aimed at understanding the origin and evolution of canine EnPEC are very limited and only reveal a significant phylogenetic distance from strains of different hosts such as bovines or equines (27).

Our results confirm that the EnPEC that infect the canine uterus mostly belong to the highly virulent phylogenetic group B2 (7, 11), that shows high capacity to colonize the reproductive tract of this species (28).

Many different virulence genes have been detected in the canine EnPEC (29) but none resulted specifically associated with pyometra when compared to isolates from cystitis or from normal intestine (7). The commonest virulence profile of canine and feline EnPEC showed the genes *fimA* and *papC*, involved in bacteria adherence to host cells, which is a prerequisite for successful colonization, *hlyA*, *hlyE* and *cnf1*, related to toxins production, responsible for host cell damage, *entB*, *ironN*, *irp1*, encoding iron uptake systems and then playing an important role in infection pathogenesis since iron is a critical nutrient in pathogenic bacteria (27).

We detected hemolysis in more than 70% *E. coli* isolates and CNF-positivity was associated to hemolytic traits in all except one strain, suggesting the presence of a pathogenicity-associated island comprising both genes (*hly* and *cnf*) (8). CDT, encoded by plasmids, was never detected. *E. coli* virulence characteristics did not result associated with any histopathological finding: the association between endometrial histopathological lesions and bacterial pathogenicity has been investigated in previous studies on dogs or dogs and cats with pyometra (28, 29), with inconclusive results except the observation that the isolation of *E. coli* resulted highly related to severe tissue damage in comparison with other bacteria (29). However, when considering only *E. coli*, the degree of endometrial lesions did not result related to the virulence profile, confirming our observations (29).

While neither the presence of *hlyA* nor *cnf1* significantly affected uterine histopathological characteristics, detection of the adhesin encoding gene *papC* was associated with a higher degree of tissue necrosis (28). Future research might be focused on this gene marker related to adhesion.

Rocha et al. (30) observed a low production of protease, siderophore and hemolysin from *E. coli* isolated from dog pyometra, suggesting that the expression of virulence-associated genes might be more related to host factors than to bacterial virulence traits. Notwithstanding the fact that these results could have been altered by antimicrobials administration before culture (30), host-pathogen interaction should be the target of future investigations aimed at understanding the relationship between *E. coli* and the severity of uterine lesions.

Cell invasion and intracellular persistence is one of the mechanisms used by EnPEC for infection maintenance (29). Another mechanism might consist in escaping the host defenses by inhibiting the PI3K pathway, that enables the cell to activate the mechanisms of killing (31). PI3K are enzymes that transmit important signals regulating a variety of physiological processes in virtually all tissue types studied to date; they act through the recruitment of effector proteins to membrane signaling complexes (13) and have also a fundamental role in coordinating defense mechanisms in the innate immune system (32). PI3K plays important roles in many neutrophils’ functions, it is involved in the control of the response of monocytes and macrophages to pathogens mainly by limiting proinflammatory cytokine production and enhancing the synthesis of the anti-inflammatory interleukin 10 (32). Our observations on PI3K expression, although to be considered preliminary, confirm a potential interference of the pathogen with the host cells defenses: indeed, very interestingly, the most severe tissue lesion, i.e., perforation, was present when PI3K expression was absent or low, meaning that *E. coli* may have disrupted the host defense pathway. Uterine wall perforation is an infrequent complication of pyometra: in our work, it occurred in open or closed-cervix cases, always in early diestrus, with either atrophic or hyperplastic uterine wall, but all with *E. coli* and with CNF-positive hemolytic strains.

Our preliminary observations on perforated uteri show that the impairment of the host defenses can represent an expression of *E. coli* pathogenicity that drives the infection toward severe and life-threatening conditions as peritonitis following uterine rupture.

Virulence does not mean antimicrobial resistance and, on the contrary, a reduced virulence load was observed in multidrug-resistant *E. coli* isolates (11). Notwithstanding the fact that we did not isolate multidrug-resistant strains, we observed a trend toward higher percentages of resistance in CNF-negative non-hemolytic *E. coli*. The skin/soft tissue breakpoints that we adopted, when available, should be representative of the isolated strains, which have not a urinary tract origin, as confirmed by the history of the bitches included in our study, that did not report urinary tract infections. The genetic similarity of *E. coli* isolated from pyometra and urine of the same female dog suggests a tropism to both organs (33) and EnPEC *E. coli* can show similar traits to uropathogenic strains (UPEC) (7) but the etiopathologic relationship between recurrent cystitis and pyometra remains to be demonstrated.

This study has some limitations, first the low numerosity of cases that strongly reduced the power of the statistical analysis. Some observations were incomplete, and the histological characteristics of the infected uteri are fully described in 15 out of 17 cases. A single area of uterine wall was subjected to histological analysis because numerous studies showed that uterine pathological conditions involving the whole organ, like pyometra, endometritis, cystic endometrial hyperplasia, commonly show a symmetric presentation, without differences between the left and right uterine horn (34).

Pyometra is the typical canine reproductive disease because its etiopathogenesis is strictly connected to the peculiarity of the oestrous cycle of this species, with a very long progesterone stimulation of the uterus (35). Although widely studied, many aspects of pyometra remain unknown: the severity of tissue lesions is only partly related to the virulence of *E. coli* and, when uterine wall perforation occurs, it is likely that host characteristics or the interaction between host and pathogen, with the elusion of the host defenses and the persistence of infection, play a key role. Future research can be focused on this novel aspect of the etiopathogenesis of this common condition that affects many elderly intact bitches (1).

Notwithstanding the limited number of cases, the observations reported in this study open a new perspective in the investigation on the mechanisms related to *E. coli* pathogenicity and on the possible evolution in tissue perforation leading to peritonitis as a severe complication of pyometra.

## Data Availability

The original contributions presented in the study are included in the article/Supplementary material, further inquiries can be directed to the corresponding author.
