# Supplementary material for: Endometrial pathogenic Escherichia coli in canine pyometra: severity of tissue damage, host defense evasion, and antimicrobial resistance profile
Source: Front Vet Sci. 2026 Feb 12;13:1676990. doi: 10.3389/fvets.2026.1676990 (PMC12935625; doi:10.3389/fvets.2026.1676990)

Supplementary Material

# Supplementary Material 1. Primers and Multiplex End-Point PCR Kit Instruction (used for the determination of the *E.coli* phylogenetic group)

**Quadruplex PCR for phylo-groups A, B1, B2, C, D, E (*Escherichia coli* sensu stricto) and for *Escherichia* clade I:**

| **Primer name** | **Sequence** |
| --- | --- |
| Primer AceK_f | 5’- AACGCTATTCGCCAGCTTGC -3’ |
| Primer ArpA1_r | 5’- TCTCCCCATACCGTACGCTA -3’ |
| Primer chuA.1b_f | 5’- ATGGTACCGGACGAACCAAC -3’ |
| Primer chuA.2_r | 5’- TGCCGCCAGTACCAAAGACA -3’ |
| Primer TspE4C2.1b_f | 5’- CACTATTCGTAAGGTCATCC -3’ |
| Primer TspE4C2.2b_r | 5’- AGTTTATCGCTGCGGGTCGC -3’ |
| Primer yjaA.1b _f | 5’- CAAACGTGAAGTGTCAGGAG -3’ |
| Primer yjaA.2b_r | 5’- AATGCGTTCCTCAACCTGTG -3’ |

| **Reagent** | **Initial concentration** | **Final concentration** | **µL x 1 reaction** |
| --- | --- | --- | --- |
| Ultrapure water | - | - | 14,1 µl |
| Reaction Buffer without MgCl_2_ (Roche) | 10X | 1X | 2,5 µl |
| MgCl2 (Roche) | 25 mM | 1,5 mM | 1,5  µl |
| dNTPs  (Roche) | 10 mM | 200 nM each | 2  µl |
| Primer AceK_f | 20 µM | 0,2 μM | 0,25 µl |
| Primer ArpA1_r | 20 µM | 0,2 μM | 0,25 µl |
| Primer chuA.1b_f | 20 µM | 0,2 μM | 0,25 µl |
| Primer chuA.2_r | 20 µM | 0,2 μM | 0,25 µl |
| Primer TspE4C2.1b_f | 20 µM | 0,2 μM | 0,25 µl |
| Primer TspE4C2.2b_r | 20 µM | 0,2 μM | 0,25 µl |
| Primer yjaA.1b _f | 20 µM | 0,2 μM | 0,25 µl |
| Primer yjaA.2b_r | 20 µM | 0,2 μM | 0,25 µl |
| FastStart Taq DNA Polymerase **(Roche)** | 5 U/µl | 0,08 U/µl | 0,4 µl |
| Total volume mix | - | - | 22,5  µl |
| Sample volume | - | - | 2,5  µl |
| Final reaction volume | - | - | 25  µl |

| **PHASE** | **TEMPERATURE/ TIME** | **n°**  **CYCLES** |
| --- | --- | --- |
| Initial denaturation | 94°C/ 5’ | 1 |
| Denaturation | 94°C/ 30’’ | 30 |
| Annealing | 59°C/ 30’’ |  |
| Elongation | 72°C/ 30’’ |  |
| Final elongation | 72°C/ 5’ | 1 |
| Final hold | 10°C | ∞ |

**PCR specific for phylo-group C:**

| **Primer name** | **Sequence** |
| --- | --- |
| Primer trpAgpC.1_f | 5’- AGTTTTATGCCCAGTGCGAG -3’ |
| Primer trpAgpC.2_r | 5’- TCTGCGCCGGTCACGCCC -3’ |

| **Reagent** | **Initial concentration** | **Final concentration** | **µL x 1 reaction** |
| --- | --- | --- | --- |
| Ultrapure water | - | - | 11,75 µl |
| Reaction Buffer without MgCl_2_ (Roche) | 10X | 1X | 2,5 µl |
| MgCl2 (Roche) | 25 mM | 2,5 mM | 2,5  µl |
| dNTPs  (Roche) | 10 mM | 200 nM each | 2  µl |
| Primer AceK_f | 20 µM | 0,4 μM | 0,5 µl |
| Primer ArpA1_r | 20 µM | 0,4 μM | 0,5 µl |
| FastStart Taq DNA Polymerase **(Roche)** | 5 U/µl | 0,05 U/µl | 0,25 µl |
| Total volume mix | - | - | 20  µl |
| Sample volume | - | - | 5  µl |
| Final reaction volume | - | - | 25  µl |

| **PHASE** | **TEMPERATURE/ TIME** | **n°**  **CYCLES** |
| --- | --- | --- |
| Initial denaturation | 94°C/ 5’ | 1 |
| Denaturation | 94°C/ 30’’ | 30 |
| Annealing | 59°C/ 30’’ |  |
| Elongation | 72°C/ 30’’ |  |
| Final elongation | 72°C/ 5’ | 1 |
| Final hold | 10°C | ∞ |

**PCR specific for phylo-group E:**

| **Primer name** | **Sequence** |
| --- | --- |
| Primer ArpAgpE_f | 5’- GATTCCATCTTGTCAAAATATGCC -3’ |
| Primer ArpAgpE_r | 5’- GAAAAGAAAAAGAATTCCCAAGAG -3’ |

| **Reagent** | **Initial concentration** | **Final concentration** | **µL x 1 reaction** |
| --- | --- | --- | --- |
| Ultrapure water | - | - | 13.25 µl |
| Reaction Buffer without MgCl_2_ (Roche) | 10X | 1X | 2,5 µl |
| MgCl2 (Roche) | 25 mM | 1,5 mM | 1,5  µl |
| dNTPs  (Roche) | 10 mM | 200 nM each | 2  µl |
| ArpAgpE_f | 20 µM | 0,2 μM | 0,25 µl |
| ArpAgpE_r | 20 µM | 0,2 μM | 0,25 µl |
| FastStart Taq DNA Polymerase **(Roche)** | 5 U/µl | 0,05 U/µl | 0,25 µl |
| Total volume mix | - | - | 20  µl |
| Sample volume | - | - | 5  µl |
| Final reaction volume | - | - | 25  µl |

| **PHASE** | **TEMPERATURE/ TIME** | **n°**  **CYCLES** |
| --- | --- | --- |
| Initial denaturation | 94°C/ 5’ | 1 |
| Denaturation | 94°C/ 30’’ | 30 |
| Annealing | 55°C/ 30’’ |  |
| Elongation | 72°C/ 30’’ |  |
| Final elongation | 72°C/ 5’ | 1 |
| Final hold | 10°C | ∞ |

# Supplementary Material 2. Primers and Multiplex PCR Kit instruction (used to detect the presence of virulence genes codifying Cytotoxic Necrotizing Factor - CNF and Cytolethal Distending Toxin - CDT)

| **Primer name** | **Sequence** |
| --- | --- |
| Primer CDT-B comd | 5' - TTT CCA GCT ACT GCA TAA TC - 3' |
| Primer CDT-B comu | 5' - TAA ATG GAA TAT ACA TGT YCG - 3' |
| Primer CNF Short F | 5' – GGG GAT CAG TTT TGA TCA - 3' |
| Primer CNF Short R | 5' – CMA CTT CAT AGT AGA TGC C - 3' |

| **Reagent** | | **Initial concentration** | **Final concentration** | **µL x 1 reaction** | |
| --- | --- | --- | --- | --- | --- |
| Ultrapure water | | - | - | 8 µl | |
| Multiplex PCR kit (QIAGEN) | | 2X | 1X | 12,5 µl | |
| Primer CDT-B comu | Primer mix  CDT/ CNF | 10 µM | 0,2μM | 0,5 µl | 2 µl  Primer mix |
| Primer CDT-B comd |  | 10 µM | 0,2μM | 0,5 µl |  |
| Primer CNF Short F |  | 10 µM | 0,2μM | 0,5 µl |  |
| Primer CNF Short R |  | 10 µM | 0,2μM | 0,5 µl |  |
| Total volume mix | | - | - | 22,5 | |
| Sample volume | | - | - | 2,5 | |
| Final reaction volume | | - | - | 25 | |

| **PHASE** | **TEMPERATURE/ TIME** | **n°**  **CYCLES** |
| --- | --- | --- |
| Initial denaturation | 95°C/ 15’ | 1 |
| Denaturation | 94°C/ 30’’ | 35 |
| Annealing | 57°C/ 90’’ |  |
| Elongation | 72°C/ 90’’ |  |
| Final elongation | 72°C/ 10’ | 1 |
| Final hold | 12°C | ∞ |

# Supplementary Material 3. Dilution range for tested antimicrobials


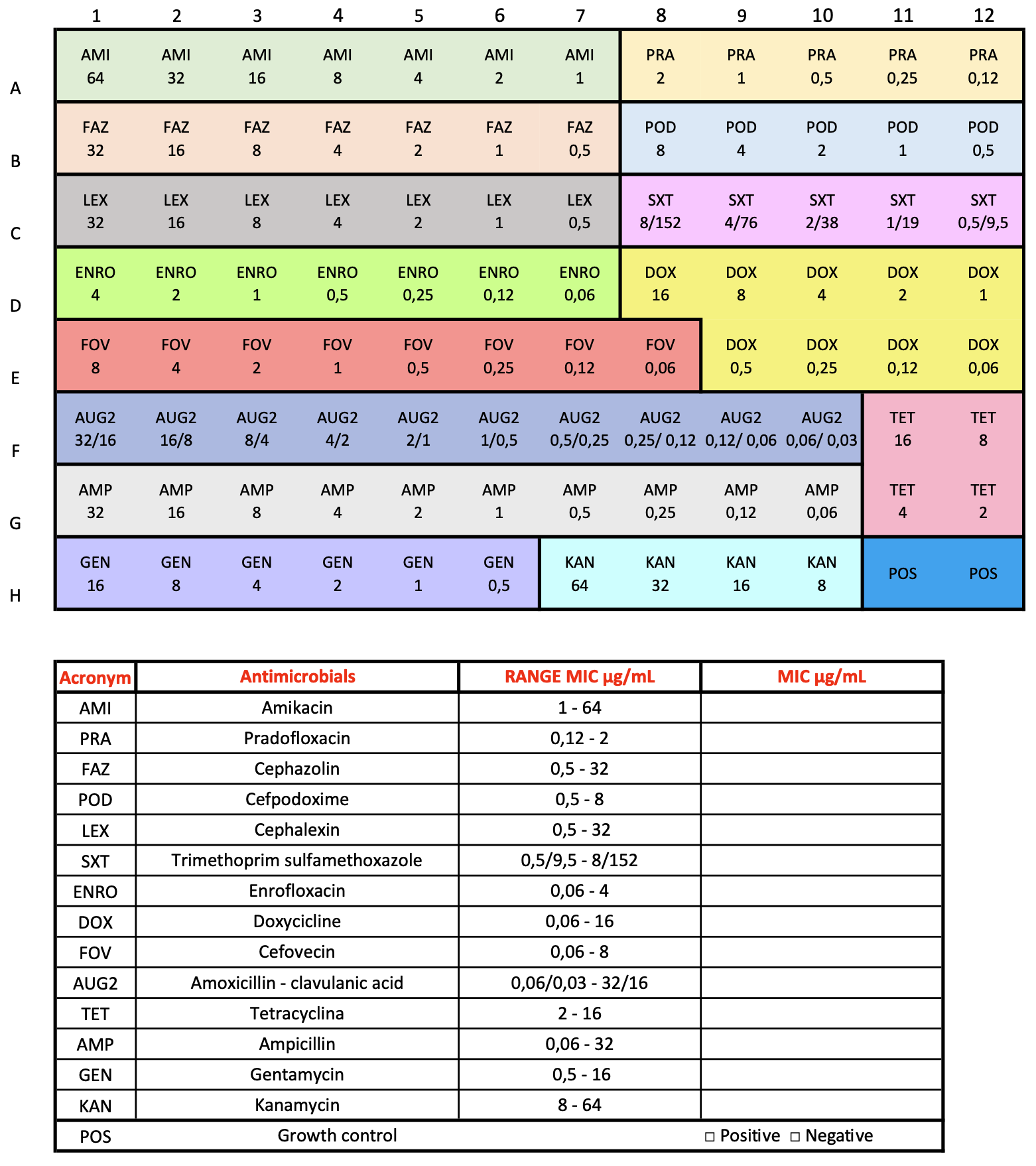

Supplement: Supplementary file 1 [file Supplementary_file_1.docx]
